# Long noncoding RNA Linc01612 represses hepatocellular carcinoma progression by regulating miR-494/ATF3/p53 axis and promoting ubiquitination of YBX1

**DOI:** 10.7150/ijbs.69514

**Published:** 2022-04-11

**Authors:** Pengpeng Liu, Qiu Zhong, Youai Song, Deliang Guo, Dong Ma, Baiyang Chen, Jianwei Lan, Quanyan Liu

**Affiliations:** 1 Department of Hepatobiliary and Pancreatic Surgery, Zhongnan Hospital of Wuhan University, Wuhan, Hubei, 430071, P.R. China.; 2 Department of General Surgery, Xiangyang Central Hospital, Affiliated with Hubei University of Arts and Science, Xiangyang, Hubei, P.R. China.; 3 Department of Hepatobiliary Surgery, Tianjin Medical University General Hospital, Tianjin, 300052, P.R. China.

**Keywords:** Hepatocellular carcinoma, Linc01612, ATF3, p53, YBX1

## Abstract

Long noncoding RNAs (lncRNAs) play an important role in the progression of hepatocellular carcinoma (HCC). Linc01612 is a novel lncRNA that function remains unknown in the progression of cancers, including HCC. In this study, we discovered that Linc01612 is significantly down-regulated in HCC tissues than in non-tumor tissues and correlated with poor prognosis. Linc01612 mainly localizes in the cytoplasm and functions as a tumor suppressor by repressing the growth and metastasis of hepatoma cells *in vitro* and *in vivo*. Mechanistically, in p53-expressing hepatoma cells, Linc01612 acts as a competitive endogenous RNA and promotes the expression of activation transcription factor 3 (ATF3) by sponging microRNA-494 (miR-494), which in turn inhibits MDM2-mediated ubiquitination of p53 and activates the p53 pathway. Furthermore, in p53-null hepatoma cells, Linc01612 exerts its biological functions by physically interacting with Y-box binding protein 1 protein (YBX1) and promoting the ubiquitin-mediated degradation of YBX1. Interestingly, the Linc01612-YBX1 signaling pathway is also present in p53-expressing hepatoma cells. In conclusion, our study indicated that Linc01612 is a functional lncRNA in HCC and Linc01612 may serve as a potential diagnostic biomarker and therapeutic target for HCC.

## Introduction

Hepatocellular carcinoma (HCC) is a common type of primary liver malignancy. Globally HCC is a major cause of cancer-related mortality especially in Africa and Asia 1-3. Hepatocellular carcinoma occurs most often in people with chronic liver diseases especially hepatitis B virus (HBV) infection 4. In the future, we will see the increase of HCC from alcoholic and non-alcoholic steatosis. Currently, the treatment of HCC is still mainly through surgery and its early intervention is the key to determine the prognosis of patients. However, the onset of HCC is usually concealed and lack of specific symptoms. Therefore, most patients are diagnosed at an advanced stage and miss the best time for treatment of HCC 5, 6. With the development of biotechnology in the recent years, there has been a more in-depth understanding of HCC. Biotechnology has brought new hope to early diagnosis and treatment of HCC especially with the emergence of various early diagnostic markers and targeted drugs of HCC cancer.

Long non-coding RNAs are a class of transcription products that are more than 200 nucleic acid bases in length and with no coding ability 7-9. Together with mRNAs, lncRNAs have several similar characteristics. For instance, they are transcribed by RNA polymerase II, and they can be capped, spliced, and polyadenylated 10. Long non-coding RNAs have been regarded as transcriptional noise 11. However, recent studies on these 'noises', have found that lncRNAs are widely expressed in various human tissues and some lncRNAs have significant differences in tumor and normal tissues. This suggests that lncRNAs may play a significant role in the progression of cancer 12. Previous studies have confirmed that lncRNAs are involved in the regulation of gene expression at the transcriptional and post-transcriptional levels 10, 13. In addition, the roles of lncRNAs are usually related to their localization in the cell. Those localized in the cytoplasm usually act as competing endogenous RNAs (ceRNAs) and affect related gene expression by sponging miRNAs. For instance, SNHG6 promotes MAT2A expression by competitive binding on miR-1297 14. The lncRNAs located in the nucleus usually influence chromatin status and regulate transcription of different genes. For example, lncRNA LCPAT1 promotes progression of breast cancer by enhancing transcription of MFAP2 gene through recruitment of chromatin remodeling factor RBBP4 to the promoter region of the MFAP2 15. Although lncRNAs have become a hot topic in cancer research, there are still many lncRNAs whose functions are completely unknown, which is worthy of further study.

Through databases analysis, we excavated a lncRNA termed Linc01612, which was significantly differentially expressed in HCC, and its potential function in HCC has not been described. In this study, we indicated that Linc01612 is downregulated in HCC tissues. Moreover, *in vitro* and* in vivo* experiments showed that Linc01612 is a key regulator of HCC growth and metastasis. Mechanistically, it was demonstrated that Linc01612 could sponge miR-494 to upregulate the expression of ATF3 and promote ATF3-mediated prevention of p53 ubiquitination. Further, in p53 defective HCC cells, Linc01612 exerts biological functions via interacting with YBX1 and promoting its ubiquitin-mediated degradation. Interestingly, the YBX1 mediated pathway is equally inhibited in p53 expressing HCC cells. Therefore, our study systematically elucidated the mechanism of Linc01612 in HCC and may provide a new possible target for HCC therapy.

## Materials and methods

### Tissue samples and clinical specimens

This study collected human HCC tissue samples and paired adjacent tissue samples from 80 patients with HCC in Zhongnan Hospital of Wuhan University. The patients included had clear pathological diagnosis and had not been treated with radiotherapy or chemotherapy before surgery. All the collected tissue samples were well preserved at -80 °C. Written informed consent was obtained from all patients, and the study was approved by the protection of human subjects committee of Zhongnan Hospital, Wuhan University. During the first 5 years of follow-up, patients were examined every 3 to 6 months and subsequently yearly. The time between primary surgery and death was deemed as the overall survival time while the duration between primary surgery and clinical disease recurrence was determined as the disease-free survival time.

### Cell culture and reagents

The human hepatocellular carcinoma cell line HCCLM3, SK-Hep1, Hep3B, the normal human liver epithelial cell line THLE-3, and the human embryonic kidney cell 293T were all cultured in DMEM (HyClone, USA) supplemented with 10% fetal bovine serum (Gibco). Cells were grown at 37° C and 5% CO2 in a humidified incubator (Thermo Fisher Scientific, USA). All the cell lines were verified by Short Tandem Repeat (STR) analysis.

### Data mining

**Figure 1:**
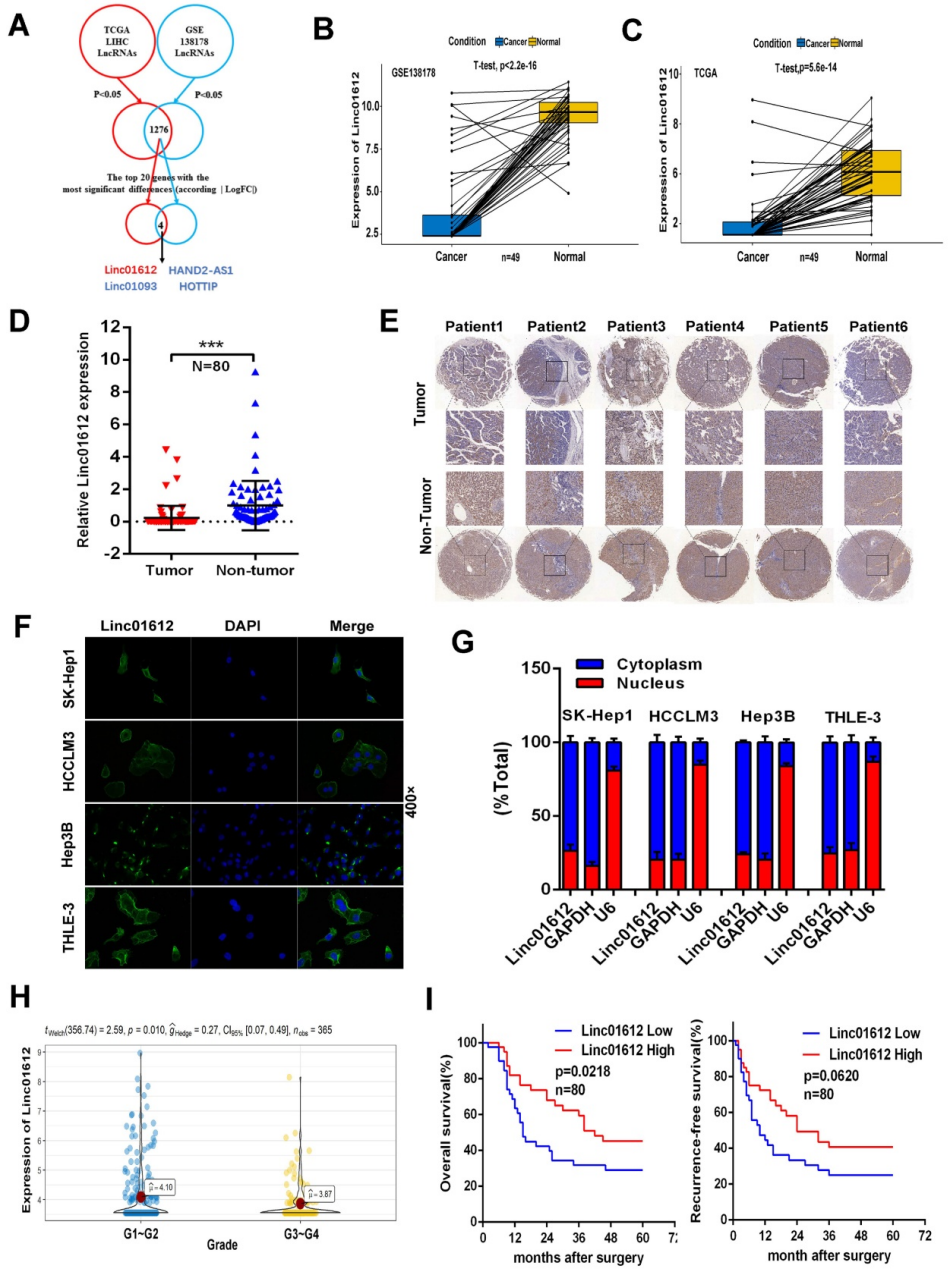
** Linc01612 is downregulated in HCC tissues and correlates with patient survival. (A)** Comprehensive analysis of GSE database and TCGA database was conducted to look for lncRNAs with significant different expression in HCC. **(B)** The expression of Linc01612 in paired HCC samples from GEO database (GSE138178). **(C)** The expression of Linc01612 in paired HCC samples from TCGA database. **(D)** The expression level of Linc01612 was lower in HCC tissues compared to paired adjacent tissues (n=80) as quantified by RT-qPCR assay. **(E)** Representative ISH image showing that Linc01612 was lower in HCC tissues compared to paired adjacent tissues. Tissue microarray are made in our own lab. The tissue microarray contained 47 pairs of HCC specimens. **(F)** Results of the FISH assay showed that Linc01612 was primarily localized in the cytoplasm. **(G)** Linc01612 was primarily localized in the cytoplasm as determined by nucleocytoplasmic separation assay. **(H)** Analysis of TCGA data showed that Linc01612 expression correlated with the pathological grade of HCC. **(I)** Kaplan-Meier analysis of overall survival and recurrence-free survival based on Linc01612 expression levels (40 patients in each group). ***p < 0.001.

By taking the intersection, we found a total of 1276 differentially expressed (p<0.05) lncRNAs in The Cancer Genome Atlas (TCGA) and the Gene Expression Omnibus (GEO) databases. Next, we analyzed the top 20 genes with the most significant differential expression among the 1276 lncRNAs according to the absolute value of logFC, and found that Linc01612, Linc01093, HAND2-AS1, and HOTTIP were significantly different in both databases. Linc01093, HAND2-AS1, and HOTTIP have been shown to play important roles in hepatocellular carcinoma 16-18, but Linc01612 in cancer has not been reported, which aroused our interest. The flowchart was shown in Fig. 1A.

### Real-Time Quantitative PCR (RT-qPCR)

Total RNA of tissue samples or cultured cells was isolated using TRIzol Reagent (Takara), and synthesize the first stand cDNA by HiScript III RT SuperMix for qPCR (+gDNA wiper) (Vazyme) according to the manufacturer's instructions. For miRNA, One Step PrimeScript miRNAcDNA Synthesis Kit (Takara) was used to reverse transcribe. RT-qPCR was performed using ChamQTM Universal SYBR® qPCR Master Mix (Vazyme). All PCR primer sequences are presented in the Table S1.

### Western blot

HCC cells were washed three times with ice-cold PBS and lysed in RIPA lysis buffer with (Beyotime, China) with 1% protease inhibitor cocktail (MedChemExpress, USA). Equivalent protein samples were separated by SDS-PAGE gel and transferred onto a polyvinylidene fluoride (PVDF) membrane (Millipore, USA). After blocking with 5% skim milk at room temperature for 1 hour, membranes were incubated with specific primary antibodies overnight at 4 °C followed by wash with TBST for 3 times, then incubated with secondary antibody of the corresponding species. Next, membranes were washed with TBST for 3 times and visualized using an enhanced chemiluminescent (ECL) detection reagent. The primary antibodies used in this study were listed in Table S2.

### Immunoprecipitation and ubiquitination assays

HCC cells were seeded in 6-well plates and transfected with HA-Ub plasmid, Flag-p53/YBX1, Linc01612/siRNA #1 and other plasmids according to the experimental design. After 48 h, the cells were incubated with 10 μM MG132 (a potent proteasome and calpain inhibitor, HY-13259, MedChemExpress) for 8 h. The total cell lysates were collected and subjected to immunoprecipitation with flag or YBX1 antibody at 4 °C overnight. Western blot analysis was performed with Anti-HA antibodies to detect the corresponding protein ubiquitination.

### Immunohistochemistry (IHC) and cell Immunofluorescence (IF)

For immunohistochemistry, the embedded-paraffin tissues were sectioned with a thickness of approximately 4-5 μm. Next, paraffin sections were deparaffinized in xylene and rehydrated with ethanol, and used 0.3% hydrogen peroxide to block endogenous peroxidase activity. Finally, the sections were incubated with specific primary antibodies and secondary antibodies. For cell Immunofluorescence, cell-planted slides were fixed with paraformaldehyde and then used 0.5% Triton X-100 to permeate for 20min at room temperature. After washing with PBS, bovine serum albumin was sealed for 1 hour, and slides were incubated with specific primary antibodies overnight at 4 °C. On the second day, the slides were incubated with fluorescent secondary antibody. Finally, nuclear staining was performed using Hochest. The primary antibodies used for IHC and IF were listed in Table S2.

### Fluorescence *in situ* hybridization (FISH)

The Linc01612 probe was designed and produced by AXL-bio (Guangzhou, China), and the specific probe sequences were listed in Table. S1. Cells were grown on 6-well plates and were treated according to previous methods 19.

### Plasmid construction and cell transfections

To overexpression of Linc01612, The full length of Linc01612 was synthesized and subcloned into the pcDNA3.1(+) vector. To knock down Linc01612 expression, three different siRNAs (Table S1) were designed and synthesized by the JTSbio (Wuhan, China). For luciferase reporter assays, Linc01612-WT or Linc0161-MUT and ATF3 3'UTR-WT or ATF3 3'UTR-MUT were synthesized and subcloned into the pmirGLO vector. For MS2-RIP assays, Linc01612-WT or Linc0161-MUT were synthesized and subcloned into the pcDNA3.1-MS2 vector. pcDNA3-flag-p53(WT), pcDNA3.1(+)-MDM2, pcDNA3.1(+)-YBX1 and pcDNA3.1(+)-myc-ATF3-his directly synthesized by the company. HA-Ub plasmid was obtained from the Department of Thyroid and Breast Surgery, Zhongnan Hospital of Wuhan University.

### Cell Proliferation Analysis

For CCK8 assay, HCC cells (1×10^4^) in 100 µl medium were seeded into 96-well plates. At different points in time, 10 μL CCK-8 solution (Dojindo, Kumamoto Ken, Japan) was added to each well and incubated at 37 °C for 2 h. Finally, the absorbance at 450 nm was measured with a microplate analyzer. For colony formation assay, the logarithmic growth cells (1×10^3^) were seeded into 6-well plates and were grown for a period of 2 weeks. Cell colonies were stabilized with 4% paraformaldehyde, stained with 0.1% crystal violet solution and manually counted.

### Wound healing Assay

In 6-well plate with marked line at the bottom, about 1×10^6^ HCC cells were seed and cultured in DMEM with 10% FBS. When cells reaching 100% confluence, wounds were made using 100 μl plastic pipette tip. Next, changed the medium to a serum-free medium. Imaged of the scratches were captured at 0 and 24 hour.

### Flow cytometry analysis

HCC Cells from different treatment groups were stained with an Annexin V-FITC/PI apoptosis kit (MultiSciences, China) according to the product specification. Next, cell apoptosis was detected by FC500 flow cytometer (Beckman-Coulter).

### Dual-luciferase reporter assay

According to the experimental design, pmirGLO plasmid and mimics were transfected into 293T cells using Lipo3000. One-day after transfection, cells were processed using the Dual Luciferase Reporter Gene Assay Kit (Beyotime, China). Next, the Fluorescence intensity was measured by a multimode reader with chemiluminescence detection function.

### RNA pulldown assay

Biotin-labeled RNA fragments were generated by *in vitro* transcription. Briefly, biotin-labeled RNAs were incubated with Hep3B cell lysates, RNA enzyme inhibitor, and streptavidin magnetic beads. After a series of washing, the RNA-protein mixture was boiled in sodium dodecyl sulfate buffer. Then, silver staining, mass spectrometry and western blot analyses were performed. The potential Linc01612-interacting proteins identified by mass spectrometry listed in Table S3.

### RNA immunoprecipitation (RIP) assay

RNA immunoprecipitation was performed using the RNA-Binding Protein Immunoprecipitation Kit (Magna RIP™) according to the manufacturer's protocol. The specific operation process referred to our previous research 14.

### *In vivo* studies

The animal studies were approved by the Institutional Animal Care and Use Committee of Wuhan University in Wuhan, China. Male BALB/c nude mice (4 weeks old) were obtained from the Vital River Laboratory Animal Technology Co. (Beijing, China). For tumor growth assays, HCCLM3 cells (about 5×10^6^) stably transfected with Linc01612 or the negative control were respectively injected into the right armpits of 5 mice, and subcutaneous tumor size was assessed every 6 days. The mice were sacrificed 30 days after injection and the xenograft tumor were removed. For the observation of lung metastases, HCCLM3 cells (about 3×10^6^, 100 μl) stably transfected with Linc01612 or the negative control were respectively injected into the tail vein of 5 mice. The mice were sacrificed 40 days after injection and the lung tissues were removed.

### Statistical analysis

GraphPad Prism 6.0 and SPSS 20.0 software were used to carry out the statistical analyses in this study. Student's t-test was performed for pairwise separation and comparison of means between different groups. Pearson chi-square tests and corrected chi-square test were performed with to obtain the clinicopathological correlations. Survival data were analyzed through the Kaplan-Meier method and log-rank test. The significant difference between different groups were reported at *p*<0.05. (*p< 0.05, **p< 0.01, and ***p < 0.001).

## Results

### Low expression of Linc01612 in HCC tissues predicts poor prognosis

Analysis of GEO and TCGA databases showed that Linc01612 was significantly down-regulated in HCC tissues (Fig. 1B and 1C). In order to verify the expression of Linc01612 in HCC, we measured the expression of Linc01612 in HCC tissues and paired adjacent tissues from the 80 patients by RT-qPCR. The results showed that the expression of Linc01612 was significant reduced in HCC samples (Fig. 1D). *In situ* hybridization (ISH) assay further confirmed the observation above (Fig. 1E). It is important to clarify the localization of non-coding RNAs for the study of their functions and mechanisms. Fluorescence* in situ* hybridization (FISH) assay and nucleocytoplasmic separation assay were performed to confirm the localization of Linc01612. The results found that Linc01612 was mainly localized in the cytoplasm not only in HCC cells (HCCLM3, SK-Hep1 and Hep3B), but also in normal cells (THLE-3) (Fig. 1F and 1G).

Subsequently, we analyzed the clinicopathological and prognostic significance of Linc01612. Analysis of pathological grading data from the TCGA database showed that patients had worse pathological grades during the low expression of Linc01612 (Fig. 1H). The analysis of the relationship between clinical characteristics and Linc01612 levels from the 80 patients according to the expression of Linc01612 (40 patients in each group), revealed that the expression of Linc01612 was significantly associated with number of tumors (*p*=0.032) and Edmondson-Steiner classification (*p*=0.030) (Table 1). In addition, the Kaplan-Meier survival analysis showed that Linc01612 could significantly enhance overall survival rate (*p*= 0.0218) but not recurrence-free survival rate (*p*=0.0620) (Fig. 1I). In conclusion, these findings implied that Linc01612 was an under expressed gene associated with patient prognosis in HCC.

### Linc01612 represses HCC cells growth and metastasis *in vitro* and *in vivo*

**Figure 2:**
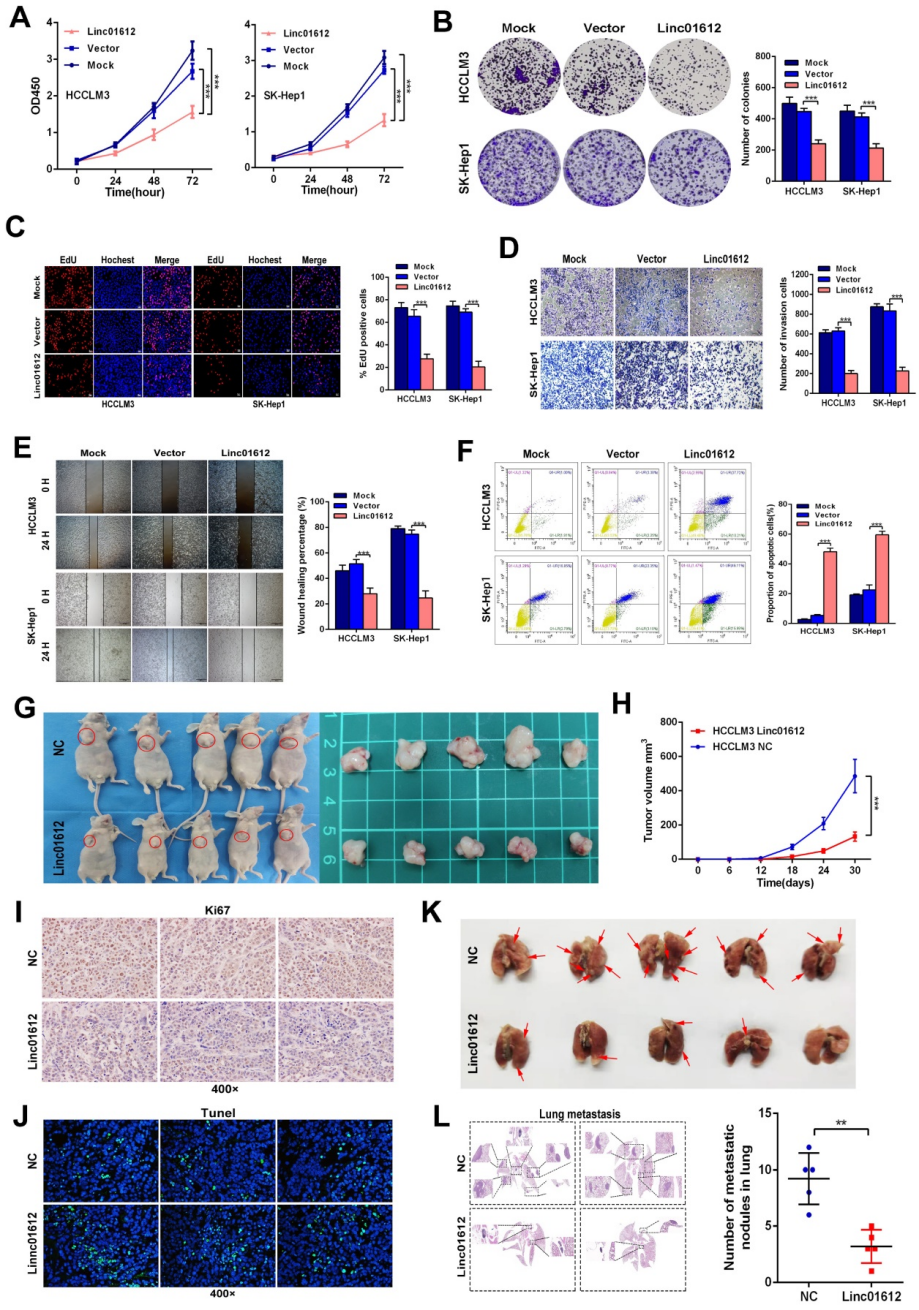
** Up-regulation of Linc01612 inhibits oncogenic behaviors *in vitro* and vivo. (A)** CCK8 assay revealed that Linc01612 inhibited the proliferation of HCCLM3 and SK-Hep1 cells. **(B)** Overexpression of Linc01612 reduced the ability of colony formation of HCCLM3 and SK-Hep1 cells. **(C)** The EDU assay showed that overexpression of Linc01612 inhibited HCCLM3 and SK-Hep1 cells growth. **(D)** Representative images of transwell assays indicated that overexpression of Linc01612 reduced HCCLM3 and SK-Hep1 cells invasion. **(E)** Representative images of wound healing assays indicated that overexpression of Linc01612 reduced HCCLM3 and SK-Hep1 cells migration. **(F)** Analysis of flow cytometric data showed that overexpression of Linc01612 promoted HCCLM3 and SK-Hep1 cells apoptosis. **(G)** Representative images of the nude mice and excised tumors that were subcutaneously injected with HCCLM3 cells stably transfected with Linc01612 or empty vector. **(H)** Analysis of tumor growth curves showed that overexpression of Linc01612 inhibited subcutaneous tumor growth. **(I)** Representative images of tumor xenografts stained with Ki-67 to examine the tumor growth. **(J) Representative** microscopic images of tumor xenografts stained with Tunel reagent to assess tumor apoptosis. **(K)** Representative images of the visible metastatic nodules in lungs. **(L)** Representative images of HE staining of metastatic nodules in lungs after different treatments and a statistical chart showing the number of lung metastases in mice. Values are expressed as the mean ± sd, n = 3 in A-F, n = 5 in H and L. **p < 0.01, ***p < 0.001.

We examined Linc01612 expression levels in different hepatoma cell lines by RT-qPCR (Fig. S1A). According to the expression of Linc01612 in HCC cell lines, we choose SK-Hep1 and HCCLM3 cells for Linc01612 overexpression, and Hep3B cells for Linc01612 knockdown. To clarify the potential function of Linc01612 in HCC, an overexpressed plasmid of Linc01612 and three siRNAs groups against Linc01612 were constructed, and the transfection efficiencies were evaluated by RT-qPCR (Fig. S1B and S1C). First, the effects of Linc01612 on tumor proliferation capacity were evaluated through the CCK8, clone formation and EDU assays. Results showed that overexpression of Linc01612 inhibits HCCLM3 and SK-Hep1 cells proliferation (Fig. 2A to 2C).

**Figure 3:**
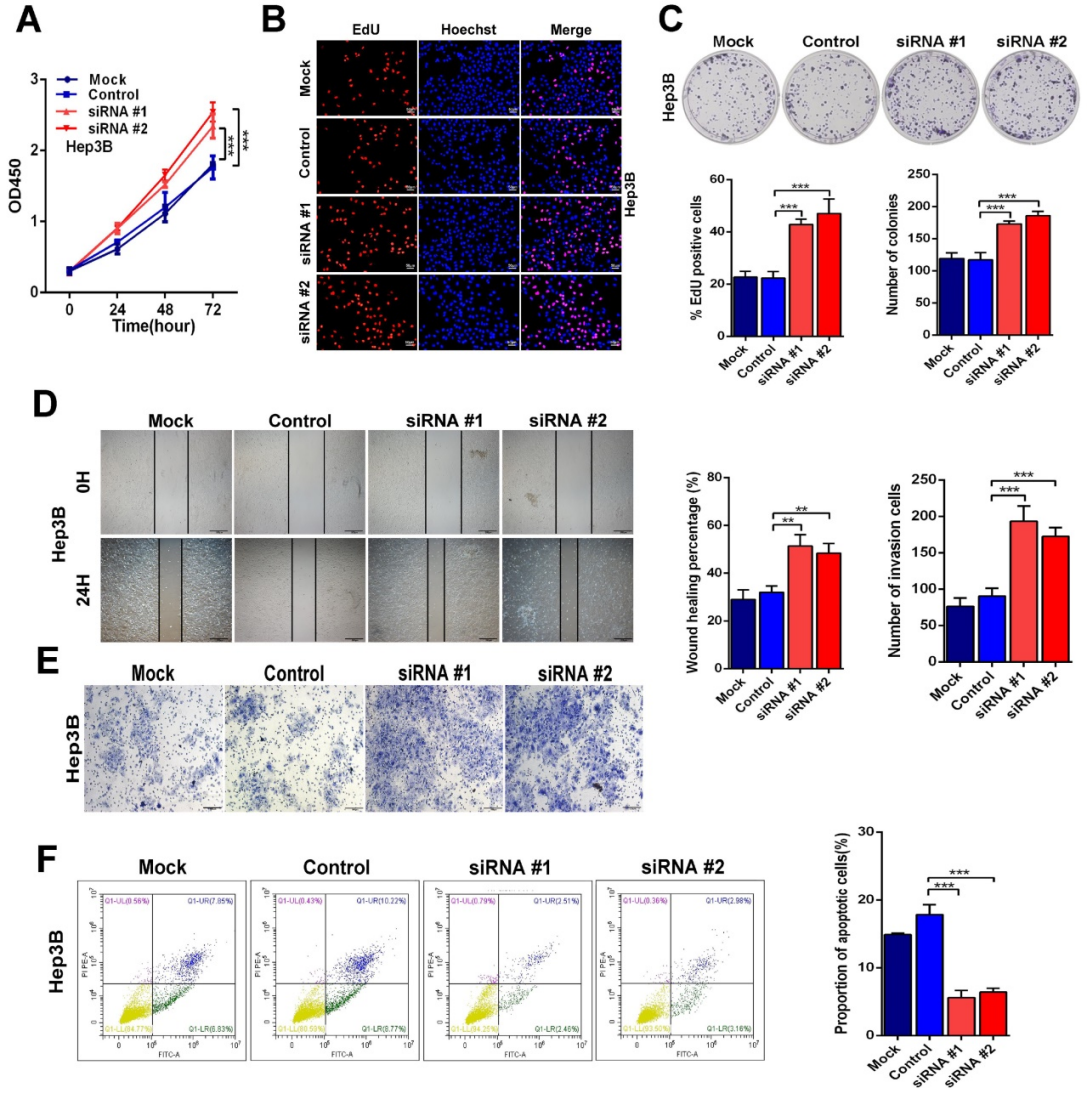
** Down-regulation of Linc01612 facilitates oncogenic behaviors *in vitro*. (A)** CCK8 assays revealed that down-regulation of Linc01612 promoted Hep3B cells proliferation. **(B)** EdU assays showed that low expression of Linc01612 promoted Hep3B cells growth. **(C)** Colony formation was increased when Linc01612 expression was decreased. **(D)** Representative images of wound healing assays indicated that low-expression of Linc01612 increased Hep3B cells migration. **(E)** Representative images of transwell assays indicated that low expression of Linc01612 increased Hep3B cells invasion. **(F)** Flow cytometric analysis determined that low-expression of Linc01612 decreased Hep3B cells apoptosis. Values are expressed as the mean ± sd, n = 3 in A-F. **p < 0.01, ***p < 0.001.

On the other hand, knockdown of Linc01612 causes contrasting effects in Hep3B cells (Fig. 3A to 3C). Secondly, the wound healing and transwell assays were performed to identify the effects of Linc01612 on tumor metastasis potential. The results indicated that overexpression of Linc01612 represses cells migration and invasion abilities in HCCLM3 and SK-Hep1 cell lines (Fig. 2D and 2E). On the contrary, knockdown of Linc01612 promotes migration and invasion in Hep3B cells (Fig. 3D and 3E). Finally, the flow cytometric analysis revealed that overexpression of Linc01612 partly facilitates apoptosis in HCCLM3 and SK-Hep1 cells (Fig. 2F). Inversely, knockdown of Linc01612 suppresses Hep3B cells apoptosis (Fig. 3F). These results indicated that Linc01612 plays a role of cancer suppressor *in vitro*.

Subcutaneous xenograft and lung metastatic models experiments were conducted *in vivo* by stably transfected with Linc01612 and empty vector in HCCLM3 cells. Compared to the negative control group, the tumor size and tumor volume were significantly decreased in Linc01612 overexpression group. These results suggested that Linc01612 has a potential inhibitory effect on the tumorigenicity of HCC cells *in vivo* (Fig. 2G and 2H). Immunohistochemistry of Ki67 and tunnel staining further confirmed the effects of Linc01612 on tumor proliferation and apoptosis (Fig. 2I and 2J). In lung metastatic models, mice in the Linc01612 overexpression group showed a decreased number of lung metastatic nodules through observation of gross specimen and a quantitative microscopic analysis (Fig. 2K and 2L). In summary, our findings suggest that Linc01612 functions as a tumor suppressor *in vitro* and *in vivo*.

### Linc01612 stabilizes p53 expression by upregulating ATF3

**Figure 4:**
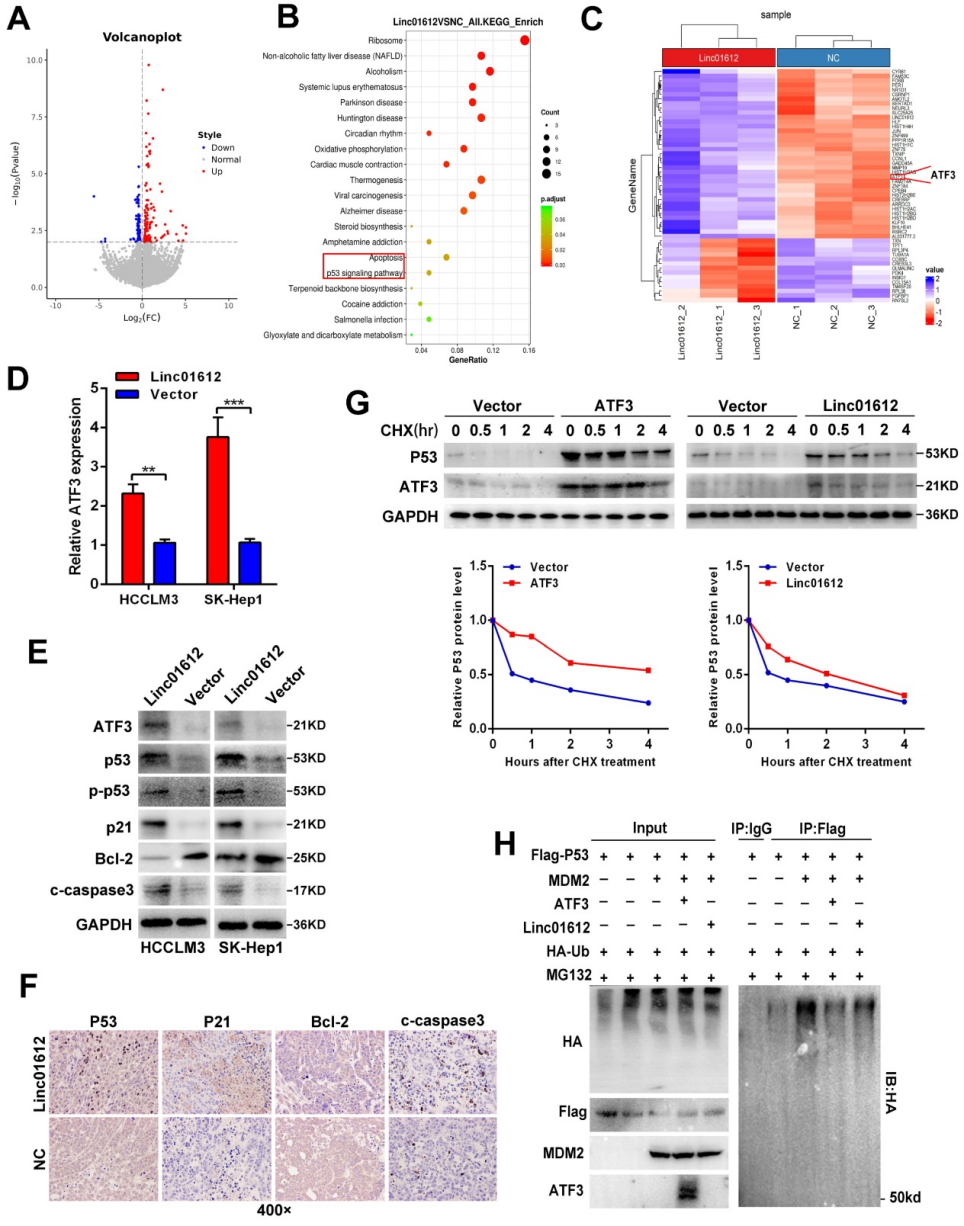
** Linc01612 down-regulates ATF3 expression and reduces p53 ubiquitin-mediated degradation. (A)** A Volcano plot showed that the differentially expressed genes between Linc01612 overexpression group and control group (fold changes >2; p<0.05). **(B)** KEGG functional enrichment analysis showed that the differentially expressed genes were enriched in apoptotic and p53 signaling pathways. **(C)** A heat map showed that the differentially expressed genes between Linc01612 overexpression group and the control group. ATF3 was upregulated in Linc01612 overexpression group. **(D)** Relative mRNA level of ATF3 in Linc01612 overexpressed and control cells. **(E)** Expression of proteins associated with p53 signaling pathway were determined by western blot. **(F)** Representative microscopic images of tumor xenografts stained with p53, p21, Bcl2 and cleaved-caspase3. **(G)** SK-Hep1 Cells co-transfected with flag-p53(WT) plasmid and ATF3/Linc01612 plasmid and then incubated with cycloheximide (CHX) for indicated time points. The protein levels of p53 were quantified by western blot (upper panel) and relative quantitative (lower panel) analysis. **(H)** Ubiquitination assay showed that overexpression of Linc01612 partially reversed P53 ubiquitination. Values are expressed as the mean ± sd, n = 3 in D. **p < 0.01, ***p < 0.001.

To find the specific mechanism of Linc01612 in HCC, we preformed transcriptome sequencing in HCCLM3 cells from different transfected groups (Fig. 4A). Kyoto encyclopedia of genes and genomes (KEGG) enrichment analysis revealed that Linc01612 was significantly associated with “Apoptosis” and “p53 signaling pathway” (Fig. 4B). The reactome enrichment analysis indicated that Linc01612 was significantly related to “Transcriptional Regulation by TP53” (Fig. S1D). Heat map analysis and differential gene expression analysis showed that ATF3, a p53 pathway correlated gene 20, was significantly upregulated after overexpression of Linc01612 (Fig. 4C and Fig. S1E). The differential expression of ATF3 was further confirmed in Linc01612 overexpressed cells (Fig. 4D and 4E) and in subcutaneous tumor tissues (Fig. S2A). In addition, bivariate correlation analysis showed that ATF3 mRNA expression was significantly positively correlated with Linc01612 transcript level in HCC tissues (Fig. S2B).

Based on the above results, we then explored the effects of Linc01612 on ATF3/p53 pathway in p53 expressing HCC cells. Western blot analysis showed that after overexpression of Linc01612 , the expression of p53, p-p53, p21, and cleaved caspase3 were enhanced whereas the expression of Bcl-2 was reduced (Fig. 4E). Similarly, xenograft tumor immunohistochemistry further found that the expressions of p53, p21 and cleaved caspase-3 were increased whereas the expression of Bcl-2 was reduced in the Linc01612-treated group compared with the control group (Fig. 4F). Considering that ATF3 could competitive activate p53 by blocking MDM2-mediated ubiquitination 20, we performed cycloheximide (CHX) chase assays in ATF3 or Linc01612 overexpressed SK-Hep1 cells. The results found that p53 stability was significantly enhanced upon overexpression of ATF3 and partly increased upon over-expression of Linc01612 (Fig. 4G). Moreover, p53 ubiquitination assays found that ATF3 observably inhibited p53 ubiquitination and Linc01612 partly blocked p53 ubiquitination catalyzed by MDM2 (Fig. 4H). Lastly, the functional recovery experiments were involved to investigate whether Linc01612 exerted its function through ATF3. Small interfering RNAs specific to ATF3 were synthesized on the basis of the previous study 21. The siRNAs knock down effects were shown in Fig. S3A and S3B. Further, the flow cytometric analysis revealed that knockdown of ATF3 could partially reverse the Linc01612 mediated increase of apoptotic cells (Fig. S3C). Cell counting kit 8 (CCK8) assays showed that knockdown of ATF3 could partially reverse the Linc01612 mediated inhibition of HCC cells proliferation (Fig. S3D). Therefore, our results indicated that Linc01612 plays an anticancer role by up-regulating ATF3 and promoting ATF3-mediated prevention of p53 ubiquitination and degradation in p53 expressing HCC cells.

### Linc01612 upregulates expression of ATF3 by sequestration of miR-494

**Figure 5:**
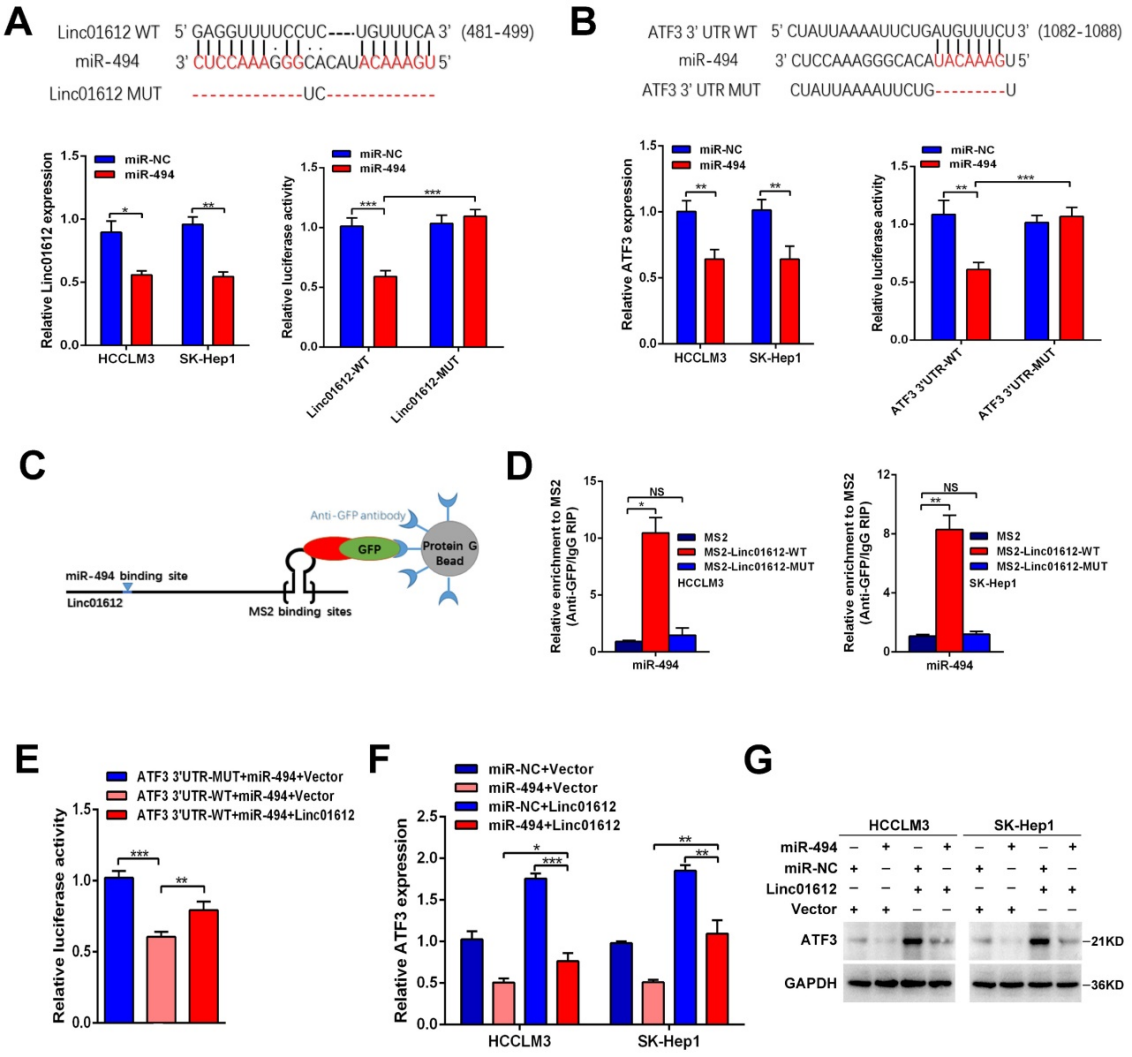
** Linc01612 upregulates ATF3 expression by sponging miR-494. (A)** Linc01612 acted as a sponge of miR-494. Wild type and mutant Linc01612 sequences were cloned into pmirGLO dual luciferase reporter vector (upper panel). Relative expression of Linc01612 in miR-494 overexpressed and control cells (lower left panel). Relative luciferase activity in miR-494 overexpressed and control cells (lower right panel). **(B)** The results showed that miR-494 bound to the 3'UTR of ATF3. Wild type and mutant 3'UTR of ATF3 sequences were cloned into pmirGLO vectors (upper panel). Relative expression of ATF3 in miR-494 overexpressed and control cells (lower left panel). Relative luciferase activity in cells overexpressing miR-494 and control cells (lower right panel). **(C)** A schematic outline of the MS2-RIP strategy. **(D)** MS2-RIP was performed for HCCLM3 and SK-Hep1 cells followed by RT-qPCR to validate the direct binding between Linc01612 and miR-494. **(E)** Relative luciferase activity in miR-494, ATF3 3'UTR, and Linc01612 co-transfected cells. **(F)** Relative mRNA expression of ATF3 in different groups. **(G)** ATF3 protein expression in different groups were determined by western blot. Values are expressed as the mean ± sd, n = 3 in A-F. *p < 0.05, **p < 0.01, ***p < 0.001.

Recent studies have shown that lncRNAs often sponge many different types of miRNAs, acting as competing endogenous RNAs (ceRNAs) to achieve their function 22-24. Because Linc01612 is mainly located in the cytoplasm, it is suspected that Linc01612 may function as a ceRNA to recover ATF3 expression by sponging miRNAs in HCC progression. The DIANA LncBase Predicted v.2 25 and TargentScanHuman 26 were used to predict Linc01612-miRNA interactions and ATF3 3'UTR-miRNA interactions respectively. The results show that Linc01612 and ATF3 3'UTR region both contain a potential binding site for miR-494. Significantly, miR-494 was identified as a cancer-promoting gene in HCC 27-29, and ATF3 was significantly downregulated in HCC tissues in TCGA LIHC dataset (Fig. S3E). Meanwhile, bivariate correlation analysis showed that ATF3 mRNA expression was negatively correlated with miR-494 expression level in HCC tissues (Fig. S2C). To verify the prediction results, this study first investigated the effects of miR-494 on Linc01612 and ATF3 expression. The results showed that overexpression of miR-494 suppresses the expression of Linc01612 (Fig. 5A lower left panel) and the expression of ATF3 (Fig. 5B lower left panel). However, Linc01612 does not seem to affect the expression of miR-494 (Fig. S2D). The full-length Linc01612 and its mutant were subcloned into the pmirGLO dual luciferase reporter vector (Fig. 5A upper panel). Dual luciferase assay showed that miR-494 significantly inhibited Linc01612 luciferase activity in wild type of miR-494 binding motif compared with the mutant type (binding motif was deleted) of miR-494 binding motif (Fig. 5A lower right panel). Luciferase reporter vectors containing the ATF3 3'UTR and its mutant were constructed synchronously (Fig. 5B upper panel). It was found that miR-494 significantly inhibited the wild type of ATF3 3'UTR luciferase activity compared with the mutant type of ATF3 3'UTR (Fig. 5B lower right panel). To further confirm that Linc01612 directly binds to endogenous miR-494, we performed MS2-RIP to pulldown endogenous miRNAs associated with Linc01612 in HCCLM3 and SK-Hep1 cells. The results indicated that miR-494 was enriched by wild type of Linc01612 (Fig. 5D). Based on such findings, we deduced that Linc01612 affects the expression of ATF3 through competitive binding of miR-494. The Linc01612, miR-494, and different luciferase reporter vectors of ATF3 3'UTR were first transfected into 293T cells to confirm the speculation. The luciferase assays revealed that overexpression of Linc01612 can partially reverse the miR-494 mediated weaken of ATF3 3'UTR-WT luciferase activity (Fig. 5E). The expression of ATF3 was later detected in different treatment groups. The results showed that miR-494 and Linc01612 co-transfection can partly enhance the expression of ATF3 compared with miR-494 and vector co-transfection (Fig. 5F and 5G). Therefore, these results suggest that Linc01612 could act as a miR-494 “trap” and relief miR-494-mediated ATF3 decay.

### Interaction between Linc01612 and YBX1

**Fig 6:**
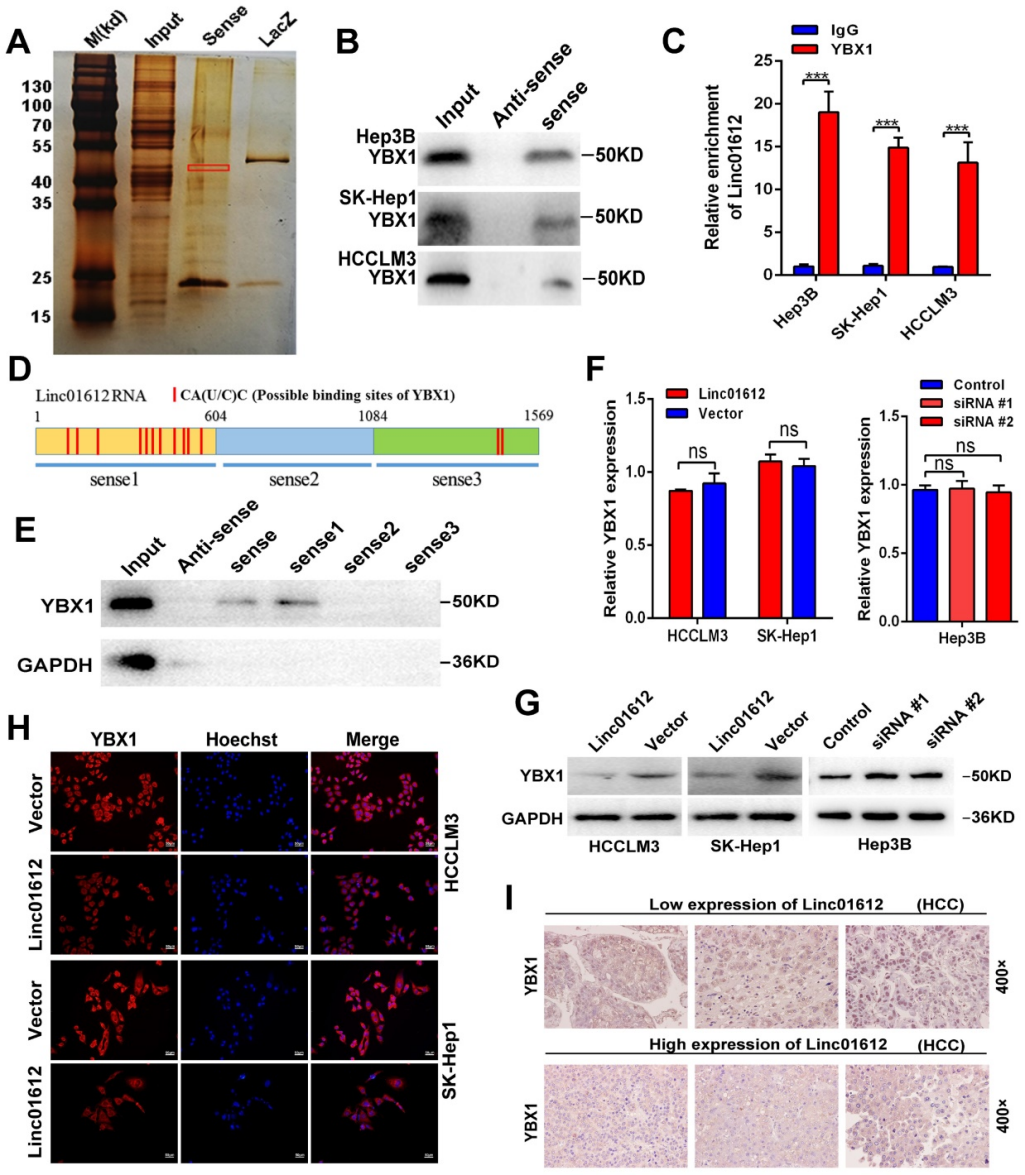
** Interaction between Linc01612 and YBX1. (A)** RNA pull down assay was performed using Linc01612 and control LacZ RNA , followed by silver staining of protein extract from Hep3B cells. **(B)** Western blot analysis of the RNA-pulldown products was performed to verify the interaction between Linc01612 and YBX1. **(C)** RIP-PCR experiments were conducted to validate the interaction between Linc01612 and YBX1. **(D)** Schematic of predicted YBX1 binding sites within Linc01612 was based on the RBPmap analysis. **(E)** Western blot analysis of YBX1 in different RNA-pulldown products induced by anti-sense Linc01612, full-length biotinylated-Linc01612 or truncated biotinylated-Linc01612 fragments (sense1: 1-604nt; sense2: 605-1084nt; sense3:1085-1569nt). **(F)** Overexpression or knockdown of Linc01612 had no significant effect on YBX1 mRNA levels. **(G)** YBX1 protein expression in different groups was determined by western blot. **(H)** Representative immunofluorescence images showed that upregulation of Linc01612 reduced YBX1 expression. **(I)** Immunohistochemical (IHC) analysis indicated that the expression of Linc01612 was negatively correlated with YBX1 expression. Values are expressed as the mean ± sd, n = 3 in C and F. ***p < 0.001.

The previous results confirmed that Linc01612 can exert tumor suppressive effect through miR-494 /ATF3/p53 axis. However, in p53 defective Hep3B cells 30, Linc01612 also function as a tumor suppressor gene (Fig. 3A to 3F). This indicates that Linc01612 might play a tumor suppressor role in another way besides p53 pathway. Recent findings have reported that lncRNAs can interact with RNA-binding proteins to perform their functions 31-33. RNA-pull down assay was performed in Hep3B cells to investigate the potential binding proteins for Linc01612 in HCC cells (Fig. 6A). Results of mass spectrometry found that YBX1 was an important binding protein for Linc01612 in Hep3B cells (Table S3). Interestingly, western blot assays further confirmed that Linc01612 can interact with YBX1 in a variety of HCC cells, not only in Hep3B cells (Fig. 6B). To validate the specific interaction between Linc01612 and YBX1, RNA immunoprecipitation (RIP) assay was carried out, and its results demonstrated that Linc01612 was enriched in YBX1 binding (Fig. 6C). To further find out the specific domain of Linc01612 which may be combined with YBX1, this study constructed three different deletion fragments of Linc01612 based on the secondary structure of Linc01612 that was predicted from the RNA fold Web server and the possible binding sites of YBX1 that was predicted from the RBPmap website 34,35 (Fig. S4A and Fig. 6D). The RNA pull-down assay was later performed using these three deletion fragments. The results showed that the region 1-604nt was likely to be the specific binding domain of Linc01612 and YBX1 (Fig. 6E).

YBX1, also known as YB-1, is highly associated with the progression and drug resistance of various cancers and can interact with a variety of non-coding RNAs to perform its biological functions 36, 37. According to the analysis of gene expression and pathological grading data obtained from TCGA database (through GEPIA2 website), we found that YBX1 was significantly upregulated in HCC tissues and associated with poor prognosis (Fig. S4B and S4C). These results were in consonance with the findings of the IHC assays (Fig. S4D). Next, we detected the effect of Linc01612 on YBX1 expression levels. Transcriptome sequencing showed that there was no significant difference in the expression level of YBX1 between the Linc01612 overexpression group and the control group (result not shown). Linc01612 had no effect on the transcriptional level of YBX1 was further confirmed by the RT-qPCR (Fig. 6F). However, the western blot assays and immunofluorescent staining results indicated that enhancing Linc01612 remarkably decreased the protein levels of YBX1 (Fig. 6G and H, Fig. S2A). In contrast, knockdown of Linc01612 upregulated the protein expression of YBX1 (Fig. 6G). Moreover, the results of IHC assays also showed that HCC tissues with low expression of Linc01612 usually have high expression of YBX1, while the high expression of Linc01612 usually have low expression of YBX1 (Fig. 6I). Collectively, these results further supported the correlation between Linc01612 and YBX1.

### Linc01612 mediates the ubiquitination and degradation of YBX1

**Figure 7:**
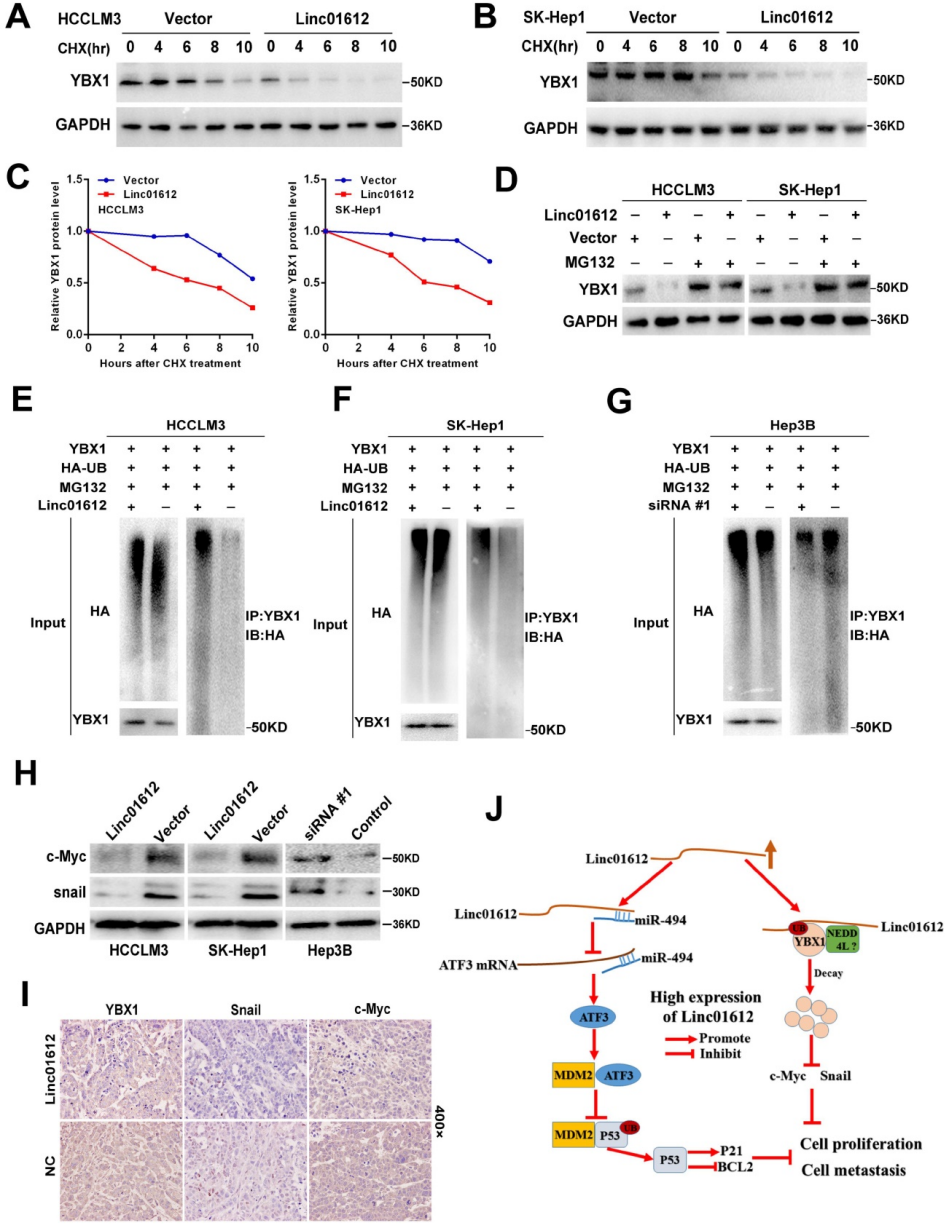
** Linc01612 downregulates YBX1 protein by inducing its ubiquitination. (A)** HCCLM3 cells transfected with Linc01612 or empty vector were incubated with cycloheximide (CHX) for the indicated time points. The protein levels of YBX1 were measured by western blot. **(B)** SK-Hep1 cells transfected with Linc01612 or empty vector were incubated with cycloheximide (CHX) for the indicated time points. The protein levels of YBX1 were quantified by western blot. **(C)** Relative quantitative analysis of YBX1 protein. **(D)** Western blot analysis of YBX1 in HCCLM3 and SK-Hep1 cells transfected with or without Linc01612 following MG132 treatment (10 µM) for 8 h. **(E), (F)** Ubiquitination assay showed that overexpression of Linc01612 partially promoted YBX1 ubiquitination. **(G)** Ubiquitination assay showed that knockdown of Linc01612 partially inhibited YBX1 ubiquitination. **(H)** Western blot analysis of the c-Myc and Snail protein levels in Linc01612 upregulation and knocked-down HCC cells. **(I)** Representative images of tumor xenografts were stained with YBX1, Snail and c-Myc antibody. **(J)** A schematic illustration of Linc01612 regulatory mechanisms in repressing hepatocellular carcinoma progression.

The results of this study showed that overexpression of Linc01612 could remarkably reduce the expression of YBX1 at the protein level but not at the mRNA level, suggested that regulation of protein level may be involved in the decrease of YBX1 levels. Therefore, it is speculated that the expression levels of Linc01612 may affect the protein stability of YBX1. Cycloheximide (CHX) chase assays found that Linc01612 reduced the stability of YBX1 both in HCCLM3 and SK-Hep1 cells (Fig. 7A to 7C). Meanwhile, treatment with the proteasome inhibitor MG132 partly antagonized the attenuation of YBX1 protein levels caused by Linc01612 (Fig. 7D). These results suggest that Linc01612 may stabilize YBX1 by preventing its proteasomal degradation. Subsequent ubiquitination assays showed that overexpression of Linc01612 resulted in increased ubiquitination of YBX1 (Fig. 7E and 7F), whereas knockdown of Linc01612 decreased YBX1 ubiquitination (Fig. 7G). In addition, the expression levels of YBX1 downstream target genes were detected and the results found that c-Myc and Snail were decreased both in HCC cells and in xenograft tumors that overexpressed Linc01612 (Fig. 7H and 7I). In contrast, knockdown of Linc01612 upregulated the expression of c-Myc and Snail in Hep3B cells (Fig. 7H). And beyond that, we also found that p-YBX1 (Ser102) was downregulated in Linc01612 overexpressed HCC cells (Fig. S4E). Finally, the functional rescue experiments were performed to investigate whether Linc01612 exerted its function through YBX1. The results of clone formation assays showed that co-transfection of Linc01612 and YBX1 partly recovered the proliferation ability of hepatoma cells when compared with co-transfection of Linc01612 and vector (Fig. S4F). Further, the results of transwell assays found that co-transfection of Linc01612 and YBX1 can partly recover the invasion ability of hepatoma cells as compared with co-transfection of Linc01612 and vector (Fig. S4G). Therefore, based on these results, it is suggested that Linc01612 can repress the biological behavior of HCC cells by binding to YBX1 and affecting its stability.

## Discussion

The transcription of RNA from non-protein coding regions of the genome is one of the most important discoveries in the genomics era widespread 38, 39. Long noncoding RNAs are the main component among these non-coding RNAs. Although there have been extensive molecular studies of lncRNAs functions, the functions of the vast majority of lncRNAs still remains unidentified. In this study, through databases screening, we found a significantly differentially expressed lncRNA termed Linc01612. We discovered that Linc01612 was mainly localized in the cytoplasma and low-expressed in HCC tissues. Meanwhile, our results showed that patients with low expression of Lnic01612 had poor pathological grade and poor survival prognosis. These suggest that Linc01612 may be a potential prognostic predictor or therapeutic target of HCC. Interestingly, through analyzing TCGA database, we also found that Linc01612 showed a tendency of low expression in cholangiocarcinoma and many other cancers (Fig. S5A and S5B). Further, the expression of Linc01612 was analyzed in 8 pairs of cholangiocarcinoma samples and showed similar results (Fig. S5C). These results suggested that Linc01612 may play a key role in maintaining normal cell function. Based on the above conclusions, we have reason to believe that Linc01612 may play an important role in the progression of hepatocellular carcinoma.

In order to explore the specific mechanism of Linc01612 inhibiting the biological function of hepatocellular carcinoma, we performed transcriptome sequencing in Linc01612 overexpressed HCC cells. The functional enrichment analysis remarkably showed that the differential genes were enriched in the p53 pathway. Furthermore, the sequencing results showed that ATF3, a gene closely associated with the p53 pathway, was significantly overexpressed in the Linc01612 overexpression group. ATF3 has been shown to function as a tumor suppressor in many tumors 21, 40. The tumor suppressor activity of ATF3 is attributed to the ability to directly activate p53 in response to DNA damage 20, 41. Meanwhile, ATF3 can act as an ubiquitin "trap" to compete with p53 for MDM2-mediated ubiquitin, thereby affecting p53 expression 41. Our results finally confirmed that Linc01612 can reduce the ubiquitination level of p53 and activate p53 signal pathway by mediating high expression of ATF3. Long noncoding RNAs localized in the cell cytoplasm usually act as competitive endogenous RNAs (ceRNAs) that could bind to microRNAs through microRNA response elements (MREs) and repress microRNAs induced gene silencing 10, 42. Furthermore, in order to find out the reason why Linc01612 caused the high expression of ATF3, we predicted the miRNAs that could combine with Linc01612 and ATF3 3'UTR region through Diana LncBase Predicted v.2 and TargentScanhuman website 25, 26. Two miRNAs (miR-494 and miR-155-5p) that play a carcinogenic role in hepatocellular carcinoma caught our attention 27, 28, 43. Our research found that miR-155-5p inhibited the expression of ATF3 lesser than miR-494 (the results not shown). Meanwhile, ATF3 has been reported as a target gene of miR-494 44. Therefore, miR-494 was taken as the object of subsequent studies. Finally, we indicated that Linc01612 could interact with miR-494 to upregulate the expression of ATF3 in hepatocellular carcinoma. Although we cannot rule out the possibility that there are other regulatory mechanisms that contribute to the increased expression of ATF3 observed in Linc01612-overexpressing HCC cells, our results support the notion that Linc01612 could regulate the miR-494/ATF3/p53 axis to affect the biologic behavior of HCC cells.

Interestingly enough, this study also found that Linc01612 can play a tumor suppressor role in p53-deficient Hep3B cells. It was evident that the previous conclusion cannot explain this phenomenon. Strikingly, lncRNAs usually need to interact with one or more RNA-binding proteins (RBPs) in order to perform their biological functions 45. Does Linc01612 also work by binding to certain RNA-binding proteins? To verify this conjecture, we performed RNA-pulldown assay and mass spectrometry analysis, and the results showed that Linc01612 can interact with numerous proteins in HCC cells. Among the proteins with higher prot_score, ENO1, TUBB3, MYH9 and YBX1 were reported to promote or inhabit tumor progression by interacting with lncRNAs 46-51. LncRNA-6195 has been reported to interact with ENO1 and inhibit its enzymatic activity, thereby inhibiting the energy metabolism in HCC cells 46. LncRNA RPPH1 was identified to combine with TUBB3 and inhibit its ubiquitination, thus promoting colorectal cancer cells metastasis 47. In thyroid cancer, lncRNA PTCSC2 has been confirmed to interact with MYH9 to regulate FOXE1 expression 48. However, compared with other proteins with higher prot_score, YBX1 is the most widely studied RNA binding protein in recent years. There is growing evidence that YBX1 can interact with multiple lncRNAs and regulate tumor progression in a variety of cancers, including hepatocellular carcinoma 49-51. Meanwhile, YBX1 is closely related to the p53 pathway 51. Therefore, we focus on the interaction between Linc01612 and YBX1. Significantly, our results showed that cancer-promoting gene YBX1 could interact with Linc01612 in both p53 expressing HCC cells and p53 defective HCC cells, which demonstrated that the interaction between Linc01612 and YBX1 is universal in HCC cells. Interestingly, we found that Linc01612 had no effect on the RNA expression of YBX1, but significantly reduced the protein expression of YBX1. There are two main pathways through which intracellular protein degradation occurs, namely autophagy and the ubiquitin proteasome system. Ubiquitination proteasome system is the pathway that is more responsible for the majority of intracellular protein degradation 19. It has been reported that lncRNAs can play a role by influencing the ubiquitination of their binding proteins. For instance, lncRNA XLOC-006390 promotes the protein stability of c-Myc by blocking its ubiquitination pancreatic carcinogenesis 52. LncRNA SLC26A4-AS1 promoted DDX5 degradation via ubiquitin-proteasome pathway in thyroid cancer 53. By measuring the protein stability and ubiquitination level of YBX1 after overexpression and knockdown of Linc01612, we confirmed that Linc01612 could promote YBX1 degradation through the ubiquitin-proteasome pathway. According to Niu W,* et al*. the tumor suppressor of BRD7 can decrease the expression of YBX1 through negatively regulating YBX1 phosphorylation at Ser102 and therefore promoting YBX1 proteasomal degradation 54. This study also found that p-YBX1 (Ser102) was downregulated in Linc01612 overexpressed HCC cells. However, the underlying mechanism warrants further research. According to Huang S, *et al*. circNfix enhanced the interaction of YBX1 with NEDD4L, and induced YBX1 degradation through ubiquitination 55. Notably, the results of mass spectrometry found that Linc01612 could combine with many E3 ubiquitin ligases, such as UBE3A, UBR4, TRI14, UBR3, BRE1A, UBR5, NEDD4L, TRI41, XIAP, etc. (Table. S3), and NEDD4L is one of them. Therefore, we have reason to hypothesize that YBX1 may mediate the ubiquitination of YBX1 through NEDD4L. In addition, we acknowledge that as a non-coding RNA, Linc01612 may function by interacting with other proteins; elucidating these interactions is not the aim of the current study but warrants further research.

## Conclusion

This study identified a functionally relevant lncRNA, Linc01612, which was down-regulated in HCC tissues and associated with poor prognosis of patients. Linc01612 inhibits oncogenic behaviors both *in vitro* and *in vivo.* Mechanistically, Linc01612 exerts tumor suppressive effect by regulating miR-494/ATF3/p53 axis in p53 expressing HCC cells, and by promoting ubiquitin-mediated degradation of YBX1 in both p53 expressing and p53 defective HCC cells.

## Supplementary Material

Supplementary figures.

Supplementary table 1.

Supplementary table 2.

Supplementary table 3.

## Figures and Tables

**Table 1:** Relationship between Linc01612 expression and clinicopathologic parameters of HCC patients

Characteristics	Linc01612 expression		Number of cases	*p* value
	High (n=40)	Low (n=40)		
**Age (years)**				0.245
<55	12	17	29	
≥55	28	23	51	
**Gender**				1.000
Male	36	35	71	
Female	4	5	9	
**Tumor size**				0.172
<5	11	6	17	
≥5	29	34	63	
**Edmondson grade**				0.030*
I-II	32	23	55	
III-IV	8	17	25	
**Tumor number**				0.032*
Solitary	35	27	62	
Mutiple	5	13	18	
**HBV infection**				0.3556
Yes	36	39	75	
No	4	1	5	
**Liver Cirrhosis**				0.644
Yes	16	14	30	
No	24	26	50	
**Serum AFP(μg/L)**				0.819
<400	25	24	49	
≥400	15	16	31	
**PVTT**				1.000
Yes	4	5	9	
No	36	35	71	

PVTT: portal vein tumor thrombus; *P<0.05.

## Abbreviations

HCC: Hepatocellular carcinoma
LncRNA: Long noncoding RNA
RBP: RNA-binding protein
ceRNA: competing endogenous RNA
MREs: microRNA response elements
TCGA: The Cancer Genome Atlas
GEO: Gene Expression Omnibus database
LIHC: Liver hepatocellular carcinoma
ATF3: Activation transcription factor 3
YBX1: Y-box binding protein 1 protein
HBV: Hepatitis B virus
ISH: *In situ* Hybridization
FISH: Fluorescence *in situ* hybridization
GO: Gene Ontology
KEGG: Kyoto encyclopedia of genes and genomes
RIP: RNA immunoprecipitation
