# Supplementary material for: Long noncoding RNA Linc01612 represses hepatocellular carcinoma progression by regulating miR-494/ATF3/p53 axis and promoting ubiquitination of YBX1
Source: Int J Biol Sci. 2022 Apr 11;18(7):2932–48. doi: 10.7150/ijbs.69514 (PMC9066125; doi:10.7150/ijbs.69514)
Supplement: Supplementary file 1 — Supplementary figures. [file ijbsv18p2932s1.pdf]

Supplementary figure legends

Fig. S1: Relative expression and sequencing analysis of Linc01612. (A) Relative expression of Linc01612 in different HCC cells. (B) Relative expression of Linc01612 in transfected Linc01612 or vector HCC cells. (C) Relative expression of Linc01612 in transfected siRNAs or control Hep3B cells. (D) Reactome enrichment analysis of differentia expression genes. (E) The top ten differential genes with the most significant *P* value. ATF3 was the most significantly different gene except Linc01612. Values are expressed as the mean ± sd, n = 3 in A–C. $*p < 0.05$, $***p < 0.001$.

Fig. S2: The correlations among Linc01612, miR-494, and ATF3 levels.

(A) The expression of ATF3 and YBX1 in subcutaneous tumor tissues. (B) The correlation analysis of the Linc01612 and ATF3 expression. (C) The correlation analysis of the miR-494 and ATF3 expression. (D) The expression of miR-494 in subcutaneous tumor tissues.

Fig. S3: Linc01612 exerts tumor suppressor function through upregulation of ATF3. (A),(B) Relative expression of ATF3 in transfected siRNAs or siNC HCC cells. (C) Flow cytometric analysis revealed that knockdown ATF3 could partially reverse the Linc01612 mediated increase of apoptotic cells. (D) CCK8 assay revealed that

knockdown of ATF3 could partially reverse the Linc01612 mediated inhibition of HCC cells proliferation. (E) ATF3 was lowly expressed in non-paired HCC samples from TCGA database. Values are expressed as the mean ± sd, n = 3 in A, D and E. *p < 0.05, **p < 0.01, ***p < 0.001.

Fig. S4: Linc01612 exerts tumor suppressor function through down regulation of YBX1. (A) The predicted secondary structure of Linc01612 from RNA fold Web server (http://rna.tbi.univie.ac.at/cgi-bin/RNAfold.cgi). (B) YBX1 was highly expressed in non-paired HCC samples from TCGA database. (C) Kaplan-Meier curves for overall survival in patients with HCC from TCGA database. (D) Immunohistochemical (IHC) analysis indicated that YBX1 was upregulated in HCC tissues. (E) Western blot analysis of p-YBX1 (Ser102) in Linc01612 plasmid transfected HCC cells. (F) Colony formation assay revealed that overexpression of YBX1 could partially reverse the Linc01612 mediated inhibition of HCC cells proliferation. (G) Transwell assay showed that overexpression of YBX1 could partially reverse the Linc01612 mediated inhibition of HCC cells invasion. Values are expressed as the mean ± sd, n = 3 in A and B. ***p < 0.001.

Fig. S5: Linc01612 is differentially expressed in a variety of cancers.

(A) Linc01612 was downregulated in non-paired CHOL samples from TCGA database. (B) Linc01612 was downregulated in many cancers from TCGA database. (C) Linc01612 was downregulated in CHOL tissues compared with the paired adjacent tissues (n=8); tested by RT-qPCR. *$p < 0.05$.

Fig. S1

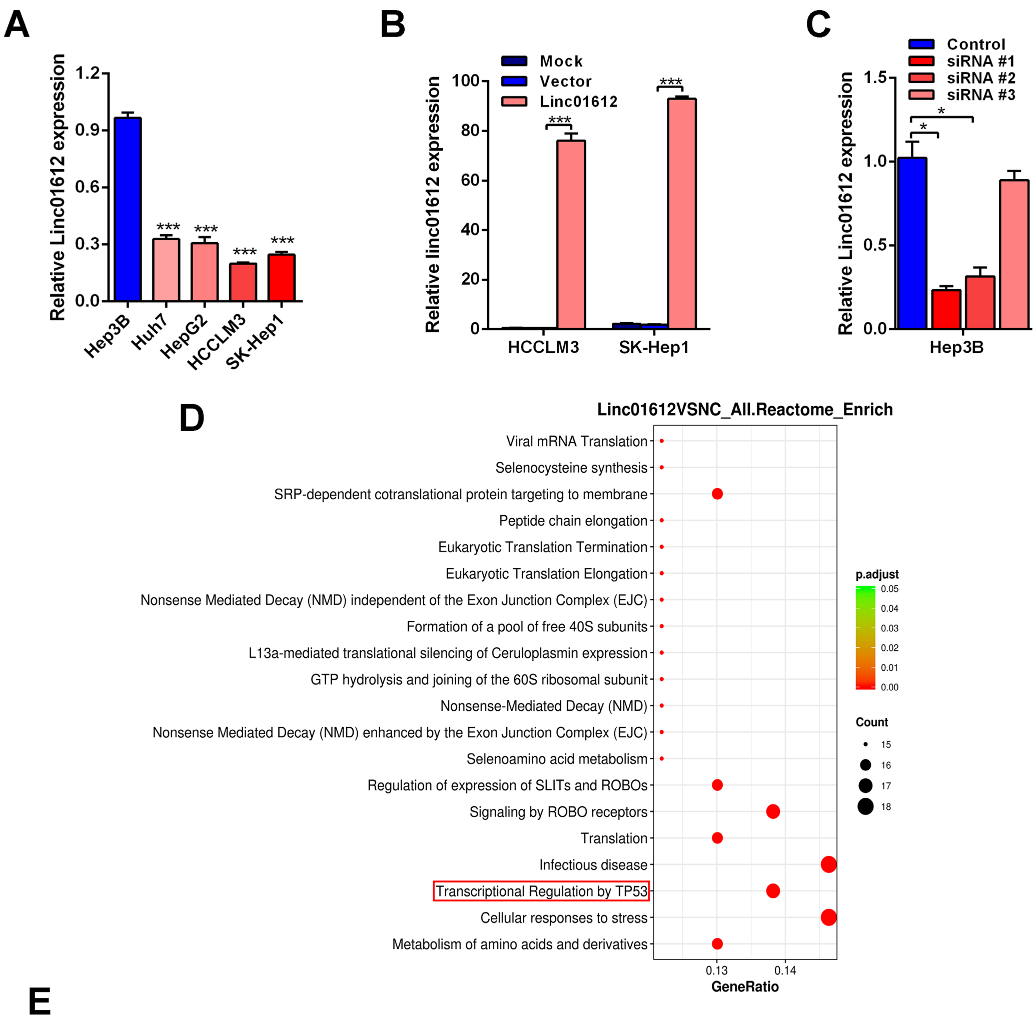

E

	A	B	C	D	E	F	G	H	I	J
1	GeneName	log2FC	Pvalue	padj	Linc01612_	Linc01612_	Linc01612_	NC_1	NC_2	NC_3
2	LINC01612	16.04114	5.39E-42	8.22E-38	23371	29658	22901	0	1	0
3	ATF3	1.158676	3.98E-23	3.04E-19	1027	1364	930	484	495	435
4	PPP1R15A	0.951647	9.14E-23	4.65E-19	2168	2620	1965	1068	1104	1159
5	NR1D1	1.187133	3.94E-19	1.50E-15	1997	2296	1706	697	945	872
6	PER1	1.618831	1.10E-18	3.37E-15	1017	1157	839	220	376	339
7	FOSB	1.37663	2.16E-15	5.49E-12	609	605	457	169	230	217
8	JUN	0.874802	3.79E-14	8.27E-11	1867	2246	1763	900	1049	1111
9	ZNF699	1.852539	2.94E-11	5.61E-08	116	163	106	31	30	40
10	HIST1H1C	0.755616	1.63E-10	2.76E-07	856	1120	896	535	512	577
11	HLF	2.388653	2.01E-09	3.07E-06	61	80	57	11	14	11

Fig. S2

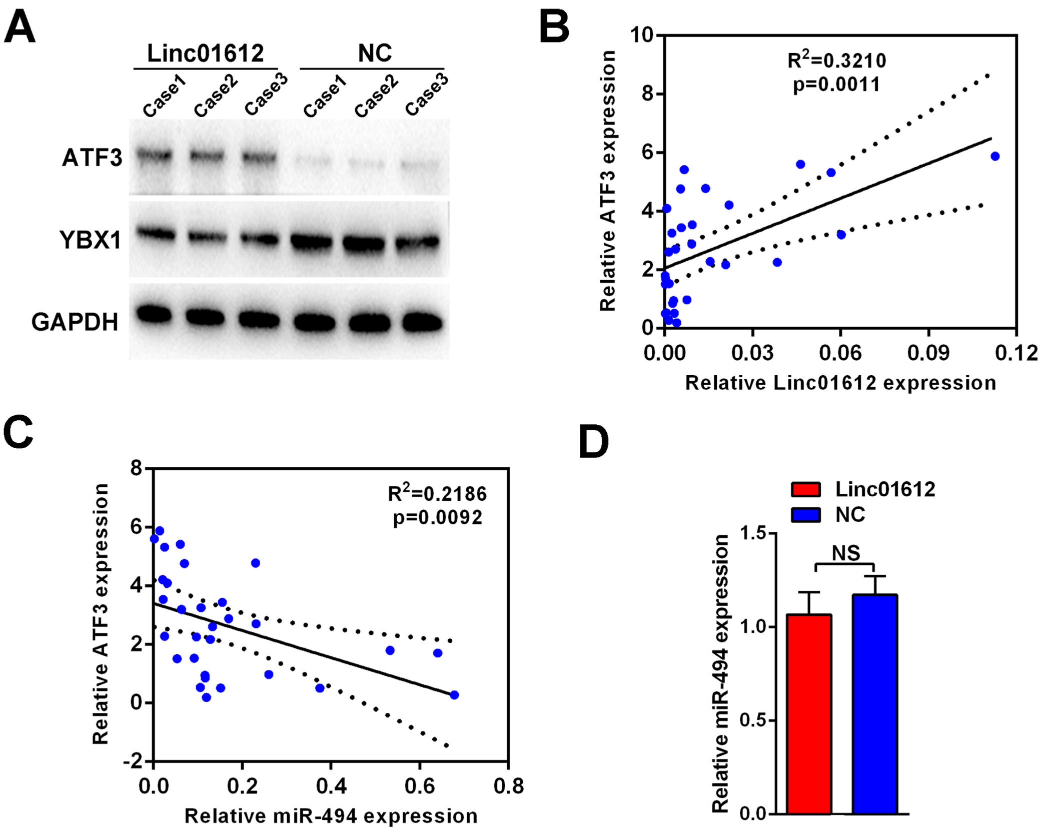

A
Linc01612
NC
Case1
Case2
Case3
Case1
Case2
Case3
ATF3
YBX1
GAPDH
B
R2=0.3210
p=0.0011
Relative ATF3 expression
Relative Linc01612 expression
C
R2=0.2186
p=0.0092
Relative ATF3 expression
Relative miR-494 expression
D
Linc01612
NC
NS
Relative miR-494 expression

Fig. S3

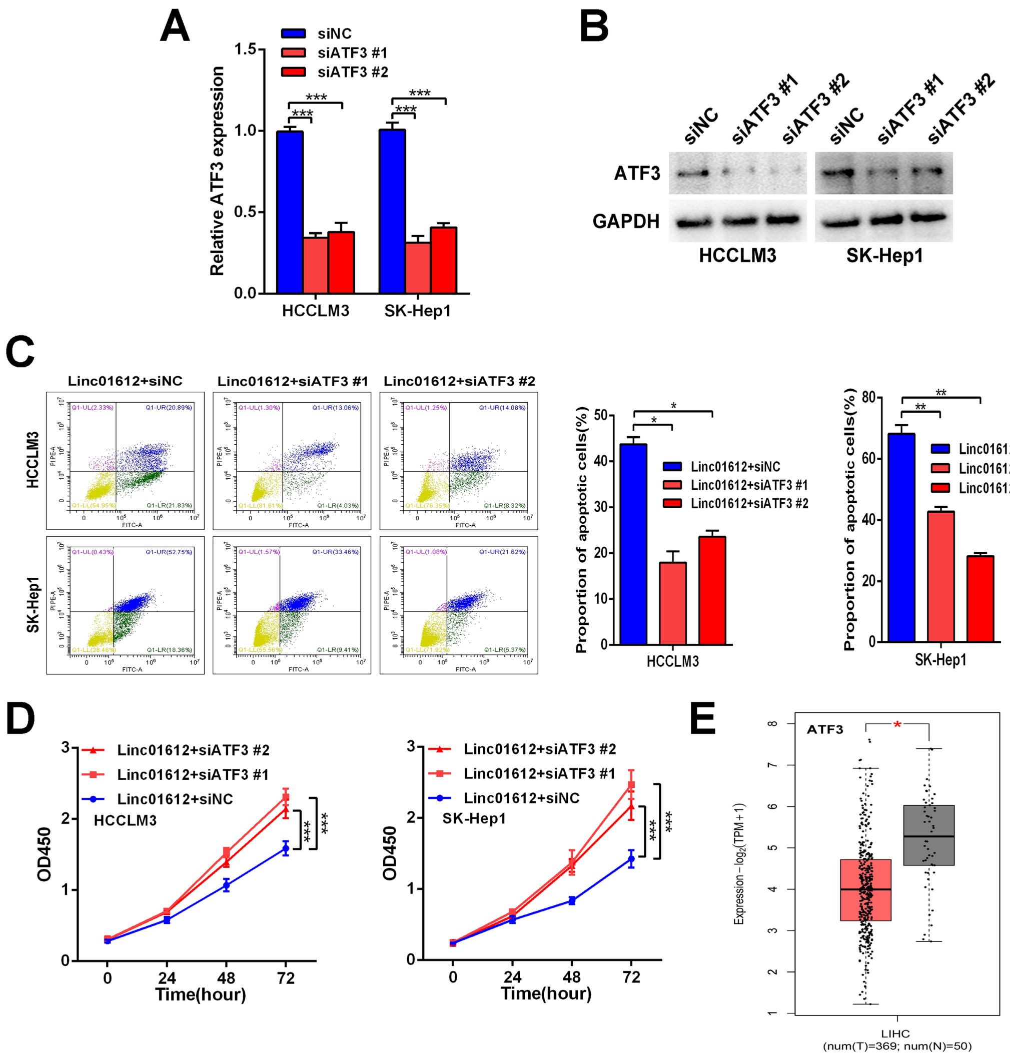
A
siNC
siATF3 #1
siATF3 #2
Relative ATF3 expression
HCCLM3
SK-Hep1
B
ATF3
GAPDH
HCCLM3
SK-Hep1
C
Linc01612+siNC
Linc01612+siATF3 #1
Linc01612+siATF3 #2
Proportion of apoptotic cells(%)
D
HCCLM3
SK-Hep1
OD450
Time(hour)
E
ATF3
LIHC
(num(T)=369; num(N)=50)

Fig. S4

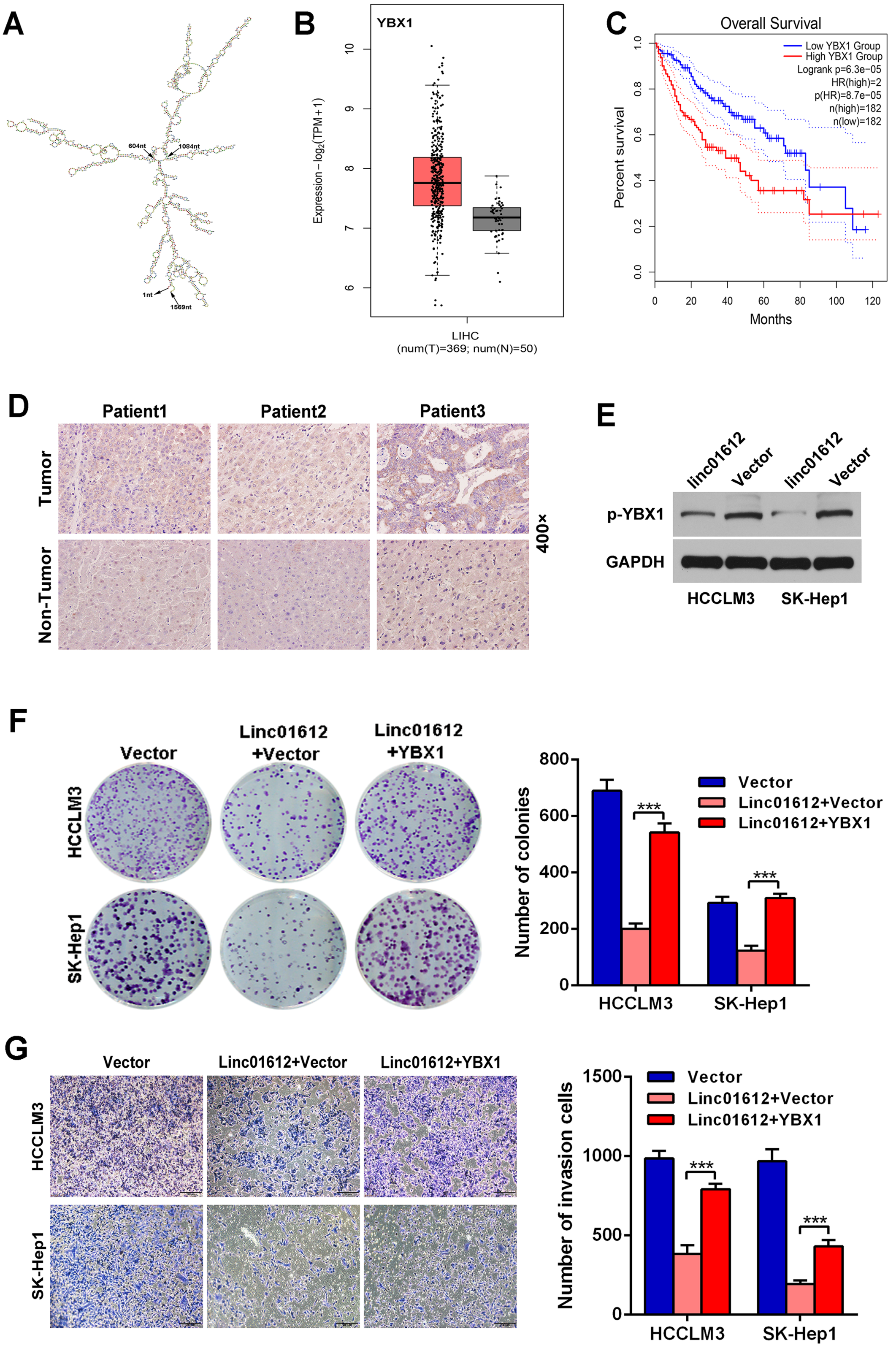

A
604nt
1084nt
1nt
1569nt
B
YBX1
Expression − log2(TPM + 1)
LIHC
(num(T)=369; num(N)=50)
C
Overall Survival
Low YBX1 Group
High YBX1 Group
Logrank p=6.3e−05
HR(high)=2
p(HR)=8.7e−05
n(high)=182
n(low)=182
Percent survival
Months
D
Patient1
Patient2
Patient3
Tumor
Non-Tumor
400×
E
linc01612
Vector
linc01612
Vector
p-YBX1
GAPDH
HCCLM3
SK-Hep1
F
Vector
Linc01612 +Vector
Linc01612 +YBX1
HCCLM3
SK-Hep1
Number of colonies
Vector
Linc01612+Vector
Linc01612+YBX1
***
HCCLM3
SK-Hep1
G
Vector
Linc01612+Vector
Linc01612+YBX1
HCCLM3
SK-Hep1
Number of invasion cells
Vector
Linc01612+Vector
Linc01612+YBX1
***
HCCLM3
SK-Hep1

Fig. S5

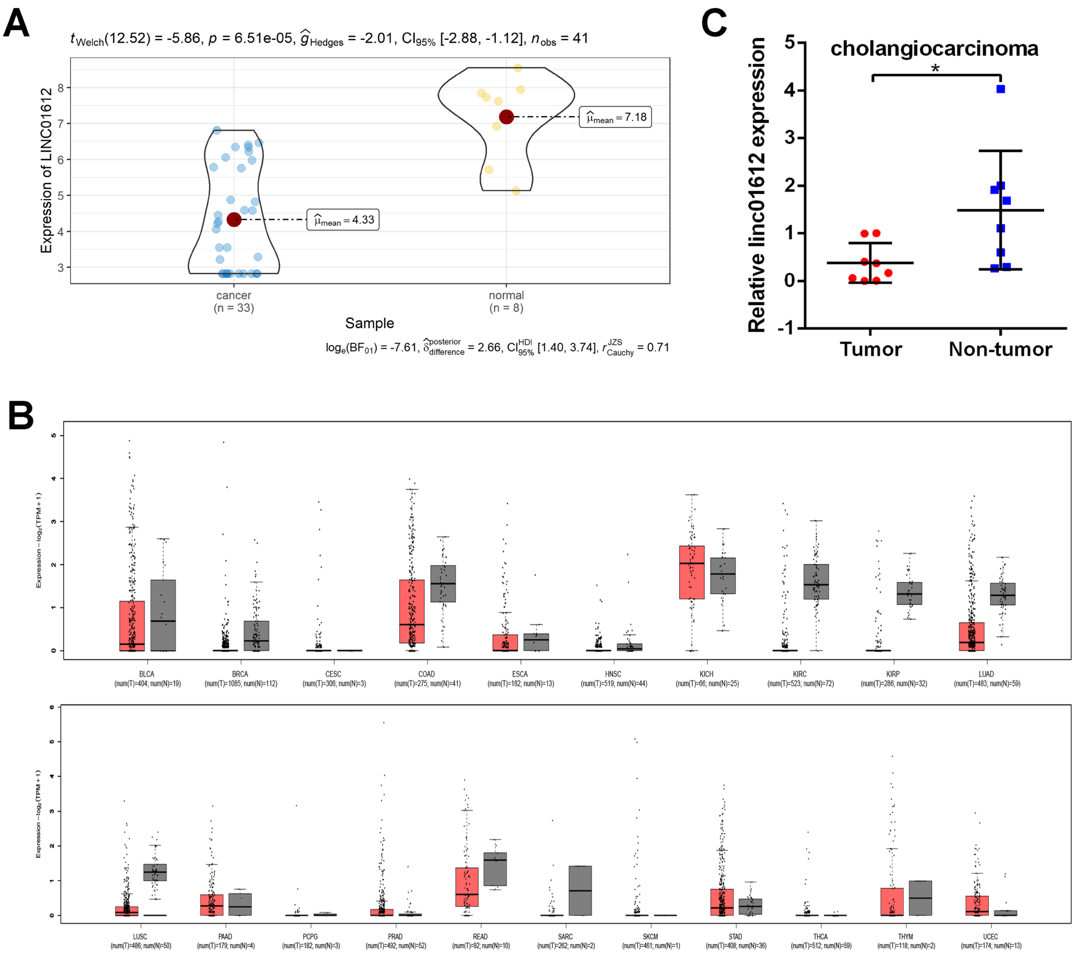
A
$t_{Welch}(12.52) = -5.86$, $p = 6.51e-05$, $\hat{g}_{Hedges} = -2.01$, $CI_{95\%}$ [-2.88, -1.12], $n_{obs} = 41$
Expression of LINC01612
$\hat{\mu}_{mean} = 7.18$
$\hat{\mu}_{mean} = 4.33$
cancer
(n = 33)
normal
(n = 8)
Sample
$\log_e(BF_{01}) = -7.61$, $\hat{\delta}^{posterior}_{difference} = 2.66$, $CI^{HDI}_{95\%}$ [1.40, 3.74], $r^{JZS}_{Cauchy} = 0.71$
C
cholangiocarcinoma
*
Relative linc01612 expression
Tumor
Non-tumor
B
BLCA
BRCA
CESC
COAD
ESCA
HNSC
KICH
KIRC
KIRP
LUAD
LUSC
PAAD
PCPG
PRAD
READ
SARC
SKCM
STAD
THCA
THYM
UCEC
